# Risk Factors and Anticoagulation Therapy in Patients With Isolated Distal Deep Vein Thrombosis in the Early Post-operative Period After Thoracic Surgery

**DOI:** 10.3389/fsurg.2021.671165

**Published:** 2021-04-28

**Authors:** Yuping Li, Junrong Ding, Lei Shen, Jian Yang, Haifeng Wang, Yiming Zhou, Gening Jiang, Yuming Zhu, Yin Wang

**Affiliations:** ^1^Department of General Thoracic Surgery, Shanghai Pulmonary Hospital, Tongji University School of Medicine, Shanghai, China; ^2^Department of Ultrasound, Shanghai Pulmonary Hospital, Tongji University School of Medicine, Shanghai, China

**Keywords:** anticoagulation therapy, IDDVT, D-dimer, thoracic surgery, risk factors

## Abstract

**Background:** Isolated distal deep vein thrombosis (IDDVT) accounts for ~50% of all patients diagnosed with deep venous thrombosis (DVT), but the diagnosis and optimal management of IDDVT remains unclear and controversial. The aim of this study was to explore potential risk factors and predictors of IDDVT, and to evaluate different strategies of anticoagulation therapy.

**Methods:** A total of 310 consecutive patients after thoracic surgery, who underwent whole-leg ultrasonography as well as routine measurements of D-dimer levels before and after surgery were evaluated. The general clinical data, anticoagulant therapy, pre- and postoperative D-dimer levels were collected. Differences between IDDVT, DVT and non-DVT groups were calculated. Logistic regression analysis was used to analyze risk factors of postoperative IDDVT.

**Results:** Age and postoperative D-dimer levels were significantly higher in IDDVT group than in non DVT group (*p* = 0.0053 and *p* < 0.001, respectively). Logistic regression analysis showed that postoperative D-dimer level was a significant independent predictor of IDDVT even when adjusted for age and operation method (*p* = 0.0003). There were no significant side effects associated with both full-dose and half-dose anticoagulation regimens. Half-dose therapy was associated with a significant decrease in the requirement for anticoagulation medications after discharge (*p* = 0.0002).

**Conclusion:** Age and D-dimer levels after surgery are strong predictors of IDDVT following thoracic surgery. Half-dose therapeutic anticoagulation has the same efficiency in preventing IDDVT progression, is not associated with any additional risks of adverse effects compared to a full-dose regimen, and may be adopted for treating IDDVT patients after thoracic surgery.

## Introduction

Deep venous thrombosis (DVT), a subset of venous thromboembolism (VTE), is a common complication of surgery with postoperative occurrence ranging from 0.7% to as high as 48% (1–4). Almost 50% of DVT patients will develop post-thrombotic syndrome symptoms, such as pain, swelling and venous ulcers in the most severe cases (5, 6). However, the key risk of untreated DVT is developing pulmonary embolism (PE) that occurs in up to one-third of cases and is the primary contributor to DVT-related mortality (7). DVTs are classified based on the anatomical site of involved venous segments. A proximal DVT may involve popliteal, femoral, and iliac veins, and the inferior vena cava (IVC) (8), while isolated distal deep vein thrombosis (IDDVT), or calf DVT, that represents around 30–50% of all lower-limb DVTs, does not extend to proximal veins (9–11). Although there is evidence that IDDVT may spontaneously resolve (12), there are reports of it extending proximally and leading to PE with a combined risk of proximal propagation and PE ranging between 7.8 and 11.4% (13–15).

Proximal DVT and PE have been extensively studied, and guidelines for their diagnosis and therapeutic anticoagulant management are well-established and optimized. According to the American College of Chest Physicians (ACCP) guidelines (16), DVT diagnosis is based on the Wells scoring system, which incorporates medical history and physical examination (17). Factors, such as advanced age, cancer, immobilization, recent trauma or surgery, and hospitalization are all considered risk factors for DVT progression into venous thromboembolism (18, 19). Risk stratification of DVT is further achieved by measuring serum levels of D-dimer, a degradation product of cross-linked fibrin. D-dimer levels are typically elevated in patients with acute thrombosis because of simultaneous activation of coagulation and fibrinolysis (20).

High risk of IDDVT proximal extension is associated with thrombosis that is extensive or close to the proximal veins, unprovoked DVT, active cancer and inpatient status (21) (Kearon). Although elevated D-dimer levels are associated with IDDVT, the usefulness of D-dimer in the diagnosis of IDDVT is limited with reported poor sensitivity (22). There have been only a few randomized clinical trials (RCTs) evaluating benefits of anticoagulation therapy for IDDVT that yielded conflicting results (14, 23–28).

One approach is to subject all the patients with suspected DVT to imaging of both the proximal and the calf veins, and to treat diagnosed IDDVT patients with anticoagulant therapy (29). Another approach uses serial imaging of proximal veins only, thus leaving IDDVT untreated (30). The need to test and treat IDDVT, therefore, remains a debated issue.

In the recent study, we explore the potential risk factors of IDDVT following thoracic surgery and evaluate optimal strategies of preventive anticoagulation therapy.

## Materials and Methods

This investigation was approved by the Institutional Review Board of Shanghai Pulmonary Hospital. Informed consent requirement was waived as this study was a retrospective study of consecutive patients that underwent thoracic surgery for diagnosed thoracic tumors of different etiology from July to November 2018. Patients were subjected to complete compression ultrasonography (31) on the 1st day after surgery.

### Patients

Study period lasted from July 2018 to November 2018. All patients that underwent thoracic surgery during this time, a total of 310 consecutive patients, were selected as potential candidates for the study. A power analysis has been performed to ensure that the number of the included patients was sufficient. Forty-seven patients were excluded due to pre-existing DVT or PTE before surgery, or missing follow up data. Based on CUS examination of the compressibility of the whole leg's veins, the remaining 263 patients were divided into non DVT and DVT groups. Of all patients with suspected DVT, 33 patients (84.6%), were further diagnosed with IDDVT based on the location of the detected thrombi. Plasma D-dimer levels of patients were assessed before and after surgery, and clinical data were collected at follow up.

### Anticoagulation Therapy

All DVT patients received anticoagulation therapy after initial diagnosis. IDDVT patients were divided into two groups. In Q12H group (*n* = 17) patients were treated with 6000 international units subcutaneous injection of low molecular weight heparin (LMWH) once every 12 h during hospitalization. In QD group (*n* = 16) patients were treated with half-dose therapeutic anticoagulation (4000–6000 IU once-daily administration of LMWH) during hospitalization.

After the discharge, patients were assessed by the physician, and the risk of VTE was determined using the Caprini risk assessment score (32). If indicated by the physician, patients continued anticoagulation treatment at home using the following regiments: Rivaroxaban (15 mg twice a day for the first 3 weeks, followed by 20 mg once a day for a total of about 3 months); Pradaxa (150 mg, twice a day for 3 months) or Aspirin (100 mg, once a day for 3 months).

Follow up ultrasound was performed 1, 3, and 6 months after discharge.

### Statistical Analysis

Statistical analyses were performed using R Software Version 3.5.3 (33). Binary and nominal discrete data was presented as counts (% of total) and comparisons were made using chi-square tests. Continuous data was presented as mean (± standard deviation) for normally distributed variables and median (IQR) for non-parametric variables and comparisons were made using two-tailed independent samples *t*-tests or Mann-Whitney *U* tests, respectively. For variables with comparisons that were significant, multiple logistic regression was used to compute odds ratios for predicting IDDVT with 95% confidence intervals to adjust for demographic and clinical variables. Among these, Age (1 case) and D-Dimer (2 cases) had missing data that were imputed with regression imputation using formulas derived from the remaining demographic variables. For all tests, *p* < 0.05 were considered statistically significant.

## Results

### Characteristics of Study Participants

**Figure 1:**
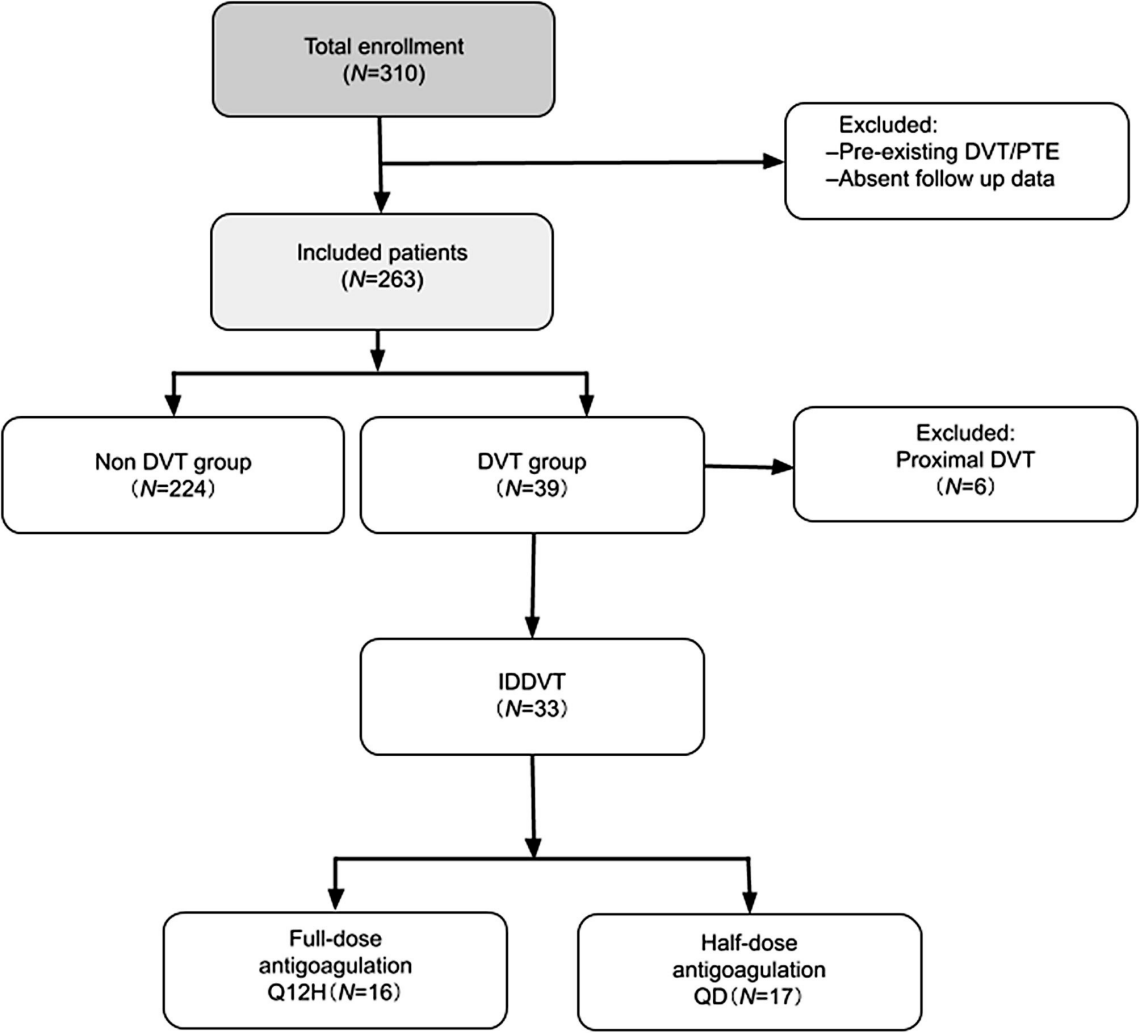
Study flow diagram. PTE, pulmonary thromboembolism; DVT, deep venous thrombosis; IDDVT, isolated distal deep vein thrombosis; Q12H, every 12 h; QD, once-daily.

Study design is shown in Figure 1. Of 331 patients considered for eligibility, 47 were excluded due to the previous history of VTE and insufficient follow up data.

Baseline characteristics of the remaining 263 patients are summarized in Table 1. The mean age of the patients was 58.8 years old, with BMI within the normal range.

**Table 1:** Baseline characteristics of patients.

	**Variables**	**Data (*N* = 263)**
Gender	Male	125 (47.6)
	Female	138 (52.4)
Age^*^	Years	58.8 (±10.1)
BMI^*^	kg/m^2^	23.5 (±2.7)
Operative	VATS	223 (84.7)
Method	Thoracotomy	40 (15.2)
Cancer	Malignant	224 (85.2)
Histology	Benign	39 (14.8)

*Data are presented as No. (%) or*

* *mean (± SD)*.

Most of the patients (224, 85.2%) were diagnosed with malignant tumors, while 39 (14.8%) were diagnosed with benign tumors. Out of 263 patients, 223 (84.7%) underwent video-assisted thoracoscopic surgery (VATS), and 40 patients (15.2%) underwent thoracotomy.

A diagnosis of lower limb DVT was established in 39 out of 263 patients (13.6%) based on the results of CUS. Comparison of baseline clinical characteristics of both DVT and non-DVT groups is shown in Table 2. DVT patients were significantly older (mean age (± SD) of 66 (±8) vs. 58 (±2) years in non DVP group, *p* = 0.001), had higher postoperative D-dimer levels (median (IQR) of 695 (1224) ng/mL as compared to 416 (353) ng/mL in non-DVT group, *p* = 0.012), and required longer hospitalization (9 (±8) days vs. 8 (±4) days in non-DVT group, *p* = 0.002).

**Table 2:** Descriptive statistics with comparison of IDDVT and non-DVT cases.

	**Unit**	**Non-DVT**	**DVT**	**IDDVT**	**DVT**	**IDDVT**
		**(*N* = 224)**	**(*N* = 39)**	**(*N* = 33)**	**vs. non-DVT**	**vs. non-DVT**
Gender^†^	Male	109 (48.7)	16 (44.4)	11 (33.3)	0.378	0.0994
	Female	115 (51.3)	23 (59.0)	22 (66.7)		
Age^‡^	Years	58 (±11.8)	64 (±8.6)	64 (±9.1)	0.002^*^	0.0053^*^
BMI^‡^	kg/m^2^	23.3 (±3.1)	23.8(±2.54)	23.7 (±2.5)	0.206	0.5307
Operation time^§^	Min	120 (86–150)	127 (102–176)	127 (105–170)	0.482	0.4777
Blood loss in surgery^§^	mL	50 (50)	50 (50)	50 (50)	0.525	0.5687
D-Dimer before surgery^§^	ng/mL	133 (96–188)	170 (103–255)	170 (103–260)	0.113	0.2150
D-Dimer after surgery^§^	ng/mL	418 (298–646)	695 (479–1,597)	705 (472–1,497)	0.001^*^	<0.0001^*^
ECG^†^	Abnormal	61 (29.3)	14 (35.5)	12 (37.5)	0.355	0.3495
	Normal	147 (70.7)	25 (64.5)	20 (62.5)		
Cancer	Malignant	189 (84.4)	35 (89.7)	30 (90.9)	0.384	0.3235
Histology^†^	Benign	35 (15.6)	4 (10.3)	3 (9.1)		
Periop. blood	Transfusion	12 (5.4)	1 (2.6)	1 (3.0)	0.732	0.5691
Transfusion^†^	None	212 (94.6)	38 (97.4)	32 (97.0)		
Operative	VATS	187 (83.5)	36 (92.3)	31 (93.9)	0.157	0.1180
Method^†^	Thoracotomy	37 (16.5)	3 (7.7)	2 (6.1)		

*All variables compared between Non-DVT (control) cases and IDDVT cases, with p < 0.05*

* * considered statistically significant. PDVT cases are displayed for reference*.

† *Binary discrete variables are presented as N (%) and compared using a chi-square test*.

‡ *Normally distributed continuous variables are presented as mean (±SD) and compared using an independent samples t-test*.

§ *Non-parametric continuous variables are presented as median (IQR) and compared using a Mann-Whitney U test*.

Of 39 patients with established DVT diagnosis, 33 (84.6%) were diagnosed with IDDVT. As summarized in Table 2, there was a significant correlation between the age of the patients and IDDVT diagnosis (*p* = 0.0053). Similarly, IDDVT patients exhibited elevated serum D-dimer levels post-surgery [median (IQR=Q3-Q1) of 705 (1025) ng/mL as compared to 418 (348) ng/mL in the non-DVP group, *p* < 0.0001]. These results suggest that age and post-operative D-dimer levels were strongly and significantly associated with IDDVT.

Since elevated D-dimer levels are associated with various pathological conditions, including cancer, we next evaluated post-surgery D-dimer concentrations in patients, diagnosed with malignancies. As shown in Table 3, similarly to the general patient population, post-surgery D-Dimer was significantly higher among patients with either DVT or IDDVT outcomes (median 705 and 708, respectively) than those without DVT (*p* < 0.0001).

**Table 3:** Comparison of post-surgical D-Dimer between DVT, IDDVT, and non-DVT cases among total cancer patients (*N* = 223).

	**Unit**	**Non-DVT**	**DVT**	**IDDVT**	**DVT vs. non-DVT**	**IDDVT vs. non-DVT**
		**(*N* = 188)**	**(*N* = 35)**	**(*N* = 30)**		
D-Dimer after surgery^§^	ng/mL	415 (299–651)	705 (501–1,780)	708 (479–1,646)	<0.0001^*^	<0.0001^*^

*D-Dimer test results following surgery compared between Non-DVT (control), DVT, and IDDVT cases among total cancer patients (N = 223), with p < 0.05*

* *considered statistically significant*.

§ *Post-surgical D-Dimer was a non-parametric continuous variable, presented as median (IQR), and compared using a Mann-Whitney U test*.

### D-dimer as a Predictor of IDDVT

We next performed predictive analysis of the variables associated with IDDVT incidence using logistic regression. D-dimer after surgery was used as an independent variable and subsequently adjusted for other significant and borderline-significant parameters, such as age, gender, and operative method (VATS vs. thoracotomy). As shown in Table 4, D-dimer remained a strong predictor of IDDVT when adjusted for age [OR (95% CI) 1.04 (1.01–1.09), *p* = 0.002], gender [OR (95% CI) 0.38 (0.16–0.88), *p* = 0.021] or operative method alone [OR (95% CI) 8.41 (1.25–56.75), *p* = 0.028]. When all the variables were combined in a final model, gender was no longer a significant factor, with OR (95% CI) of 0.44 (0.18–1.03), *p* > 0.05.

**Table 4:** Post-surgical D-Dimer as a predictor of IDDVT after thoracic surgery.

**Model**	**Coefficient**	**SE**	***p***	**OR (95% CI)**
D-Dimer	0.0006	0.0002	0.0007^*^	1.00 (1.00–1.00)
D-Dimer	0.0006	0.0002	0.0019^*^	1.00 (1.00–1.00)
Age	0.047	0.0203	0.0208^*^	1.04 (1.01–1.09)
D-Dimer	0.0006	0.0002	0.0008^*^	1.00 (1.00–1.00)
Age	0.0507	0.0207	0.0144^*^	1.05 (1.01–1.10)
Gender	−0.9696	0.4304	0.0243^*^	0.38 (0.16–0.88)
D-Dimer	0.0008	0.0002	0.0004^*^	1.00 (1.00–1.00)
Age	0.0466	0.0208	0.0250^*^	1.05 (1.01–1.09)
Operative method	2.1292	0.9742	0.0288^*^	8.41 (1.25–56.75)
(VATS vs. thoracotomy)				
D-Dimer	0.0008	0.0002	0.0003^*^	1.00 (1.00–1.00)
Age	0.0491	0.0209	0.0188^*^	1.05 (1.01–1.09)
Gender	−0.8302	0.4402	0.0539	0.44 (0.18–1.03)
Operative method	2.0225	1.0094	0.0451^*^	7.56 (1.05–54.64)
(VATS vs. Thoracotomy)				

*Models are calculated as odds ratios using logistic regression, with p < 0.05*

* *considered statistically significant for each coefficient. Odds ratios listed are for D-dimer test results as a predictor of IDDTV alone or adjusted for variables listed below for each model*.

### Cut-Off Value of Post-operative D Dimer

**Figure 2:**
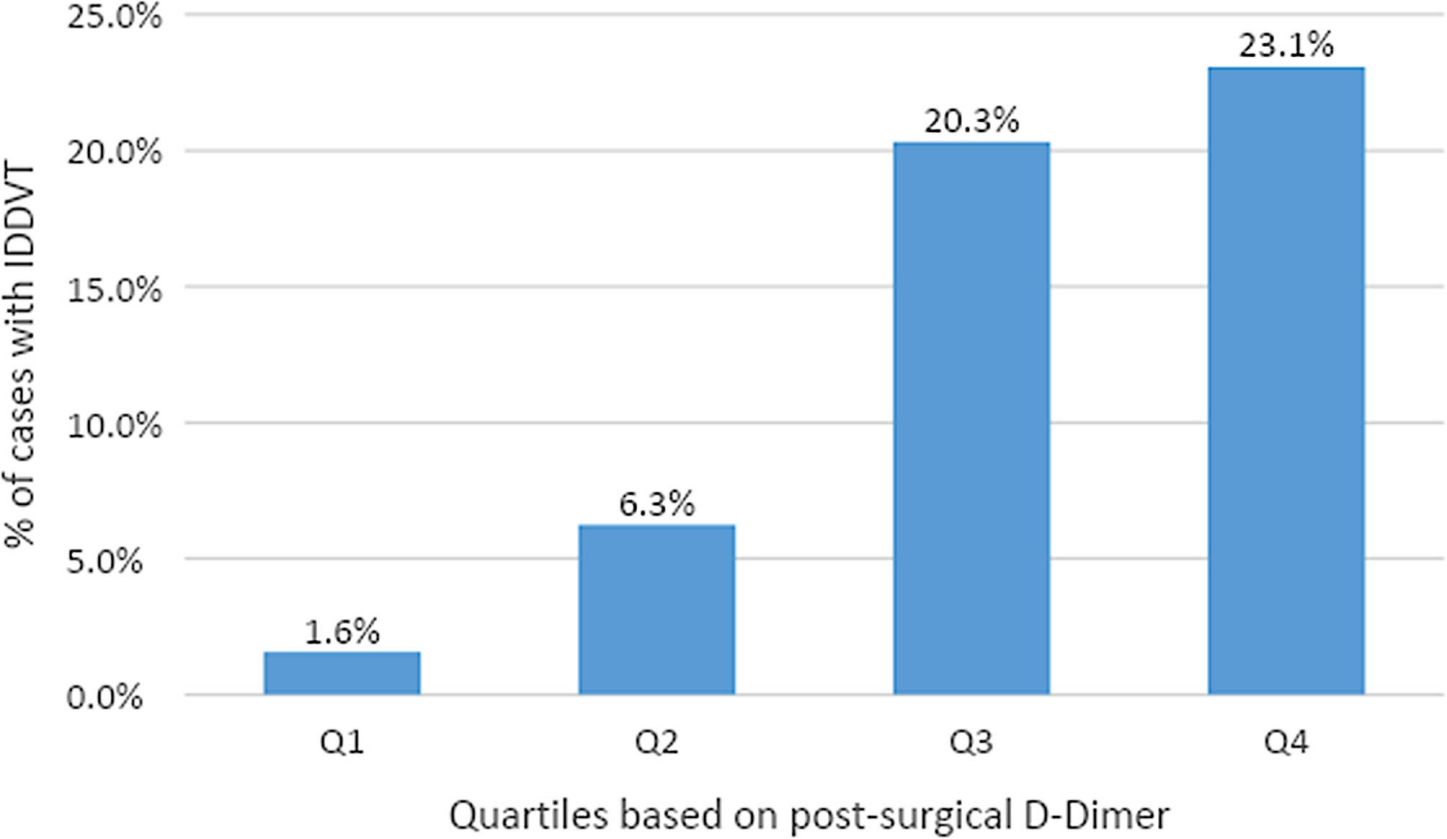
Rates of IDDVT by quartile of post-surgical D-Dimer. 1st quartile (0–306 ng/mL) had 1 case among 64 patients. 2nd quartile (306–438 ng/mL) had 4 cases among 64 patients. 3rd quartile (438–717 ng/mL) had 13 cases among 64 patients. 4th quartile (>717 ng/mL) had 15 cases among 65 patients.

We next analyzed the distribution of IDDVT rates according to post-operative D-Dimer concentrations (Figure 2). Only 1.6% of patients with D-dimer values in the lowest quartile range (0–306 ng/mL) had IDDVT diagnosis. IDDVT diagnosis rates increased to 6.3% in the second (306–438 ng/mL) quartile. The most significant rates of IDDVT were associated with the third (438–717 ng/mL) and fourth (>717 ng/mL) quartiles of D-dimer levels (20.3 and 23.1% of all cases, respectively).

The predictive effect of D-Dimer on IDDVT was further evaluated by calculating the odds ratio (unadjusted/adjusted for age and operative method) for each quartile. As shown in Table 5, cases in the lowest quartile of D-dimer have a very low risk of IDDVT, both unadjusted and adjusted [OR (95% CI) 0.08 (0.01–0.60), *p* = 0.0138, and OR (95% CI) 0.08 (0.01–0.62), *p* = 0.0157, respectively]. The risk of IDDVT increases notably in the third quartile [OR (95% CI) 2.20 (1.03–4.74), *p* = 0.0428 unadjusted, and OR (95% CI) 2.32 (1.05–5.15), *p* = 0.0379 adjusted for age and operation method]. Similar correlation between the risk of IDDVT and D-dimer concentration was observed for the fourth quartile of D-dimer values [OR (95% CI) 2.90 (1.36–6.16), *p* = 0.0056 and OR (95% CI) 2.93 (1.33–6.46), *p* = 0.0075 for unadjusted and adjusted, respectively] (Table 5). Post-operative D-dimer levels in the range of 306–438 ng/mL (second quartile) were not significant predictors of IDDVT.

**Table 5:** Quartiles of post-surgical D-Dimer as predictors of IDDVT after thoracic surgery.

**Model**	**OR (95% CI)**	***p***
D-Dimer 1st quartile^†^	0.08 (0.01–0.60)	0.0138^*^
D-Dimer 2nd quartile^†^	0.38 (0.13–1.12)	0.0784
D-Dimer 3rd quartile^†^	2.20 (1.03–4.74)	0.0428^*^
D-Dimer 4th quartile^†^	2.90 (1.36–6.16)	0.0056^*^
D-Dimer 1st quartile^‡^	0.08 (0.01–0.62)	0.0157^*^
D-Dimer 2nd quartile^‡^	0.35 (0.11–1.05)	0.0600
D-Dimer 3rd quartile^‡^	2.32 (1.05–5.15)	0.0379^*^
D-Dimer 4th quartile^‡^	2.93 (1.33–6.46)	0.0075^*^

*Models are calculated as odds ratios using logistic regression, with p < 0.05*

* *considered statistically significant for each coefficient*.

† *Unadjusted odds ratios are for each quartile of D-Dimer test results predicting IDDTV*.

‡ *Odds ratios, adjusted for age and operative method*.

### Anticoagulation Therapy

Of 33 IDDVT patients, 17 (51.5%) received a standard dose of anticoagulation therapy (Q12H group), while 16 patients (48.5%) were put on a half-dose regimen (QD group). As summarized in Table 6, there were no significant differences in outcomes between the two groups, as indicated by similar volumes of average daily drainage, requirement of chest tube indwelling, rates of thrombi recanalization and DVT progression. There were no increased safety risks, such as major bleeding, clinically relevant non-major bleeding, pulmonary embolism, or all-cause mortality associated with either of the treatment regiments. Duration of anticoagulation therapy was significantly lower in the half-dose QD group, with median (IQR) 5 (9) days compared to 99 (95) days in Q12H group, *p* = 0.004 (Table 5). Only 2 (12.5%) patients in the QD group received anticoagulation medicine after discharge, compared to 16 (76.5%) in the standard treatment Q12H group, *p* = 0.0002 (Table 6).

**Table 6:** IDDVT statistics and anticoagulation therapy follow-up results.

		**Standard Q12H**	**Half-dose QD**	**Q12H vs. QD**
		**(*N* = 17)**	**(*N* = 16)**	
	Multiple	3 (17.6)	1 (6.3)	
Duration of admission^§^	days	10 (6–12)	8 (7–12)	0.7718
Duration of anticoagulation^§^	days	99 (8–103)	5 (4–13)	0.0040^*^
Total drainage^§^	mL	620 (450–900)	720 (450–993)	0.9283
Chest tube indwelling^§^	Days	4 (3–6)	5 (4–5)	0.6384
Average daily drainage^‡^	mL	177.9 (83.6)	162.9 (91.9)	0.6271
Medication after discharge^†^	Medication	13 (76.5)	2 (12.5)	0.0002^*^
	None	4 (23.5)	14 (87.5)	
Progression of DVT^†^	Progression	0 (0.0)	0 (0.0)	N/A
	None	17 (100.0)	16 (100.0)	
Adverse events^†^	Adverse events	0 (0.0)	1 (6.3)	N/A
	None	17 (100.0)	15 (93.8)	

*All variables compared between standard therapeutic anticoagulation with 6000 IU low-molecular-weight heparin (LMWH) twice per day (Q12H) and half-dose with 6000 IU LMWH once per day (QD), with p < 0.05*

* *considered statistically significant*.

† *Binary or nominal discrete variables presented as N (%) and compared using a chi-square test*.

‡ *Normally distributed continuous variables were presented as mean (±SD) and compared using an independent samples t-test*.

§ *Non-parametric continuous variables were presented as median (IQR) and compared using a Mann-Whitney U test. Medication after discharge includes prescriptions for one of the following anticoagulants: Rivaroxaban, Pradaxa, and Aspirin. Progression of DVT includes proximal progression, progression to pulmonary embolism, or recurrent VTE. Adverse events include major bleeding, clinically relevant non-major bleeding, pulmonary embolism, or all-cause mortality (case listed was non-major bleeding)*.

## Discussion

Post-operative venous thromboembolism VTE that includes pulmonary embolism (PE) and deep vein thrombosis (DVT) is associated with a significant burden of morbidity and mortality worldwide (34). VTE leads to an 8-fold increase in mortality after general lung resection, and mortality as high as 19.8% after lung resection for cancer (35, 36). Cancer patients have a 5–7-fold increased risk of developing DVT (37, 38) and have a significantly worse prognosis, making DVT/PE one of the leading causes of morbidity and mortality in patients with cancer (39). In the current study, 224 (85.2%) of participants were diagnosed with malignant tumors. The overall incidence of DVT after surgery in our study was 14.8% (39 out 263 patients), while post-surgery DVT incidence among cancer patients was 15.6% (35 out of 224), and IDDVT incidence was 14.7%. While the average age of participants in our study was 58.8 years, incidence of postoperative DVT was associated with a significantly higher patient age (64 years in average), and 1.68-fold increase in postoperative D-dimer levels. Our results are in agreement with the previous reports that in the general population, age is a significant risk factor of DVT with patients 85 years and older having an almost 10-fold higher incidence rate of DVT/PE compared with those aged 45–54 years (40–43). The D-dimer levels as a common screening test for DVT, has been shown to be highly sensitive (>95%) in excluding acute VTE at the cutoff value of 500 ng/mL (44). However, elevated D-dimer levels are not unique to venous thrombosis, and may rise with advanced age (>65), and in various pathologic conditions including malignancy (45, 46). Our data indicate that the postoperative level of D-dimer in DVT and IDDVT patients with malignant tumors is still significantly higher (1.68- and 1.7-fold increase, respectively) than that before surgery, confirming the specific association of higher post-surgery D-dimer levels with DVT in our study.

The reports of the sensitivity and diagnostic accuracy of D-dimer level measurements for IDDVT are scarce. In a large study of 393 outpatients with clinically suspected symptomatic DVT (47–51), the D-dimer levels in patients with IDDVT were, on average, almost twice as high for patients at least 60 years old compared with those younger than 60 years (median D-dimer, 0.82 vs. 0.47). IDDVT patients in our study were older (average of 64 years as compared to 58 in non-DVT group) and had over 1.6-fold higher postoperative D-dimer serum concentrations. Since D-Dimer after surgery showed the strongest association with IDDVT incidence in our study, it was used as an independent variable, and maintained significance in all models, when adjusted for each one of the following parameters: age, gender and operative method. However, when all 4 variables were combined in a final model, gender lost its significance. Interestingly, among demographic variables in our study, gender had a borderline significant association with IDDVT (IDDTV vs. non-DTV, *p* = 0.0994). Previous studies showed that male gender was related to a higher recurrence risk of unprovoked DVT (52), but the overall role of gender in the causation of DVT is unclear. Borderline statistical significance observed may be due, therefore, to the small sample size of IDDVT patients in our study (*n* = 33).

D-dimer measurements appear to be less sensitive for the diagnosis of IDDVT than for the proximal DVT, with some IDDVT patients having D-dimer levels below the usual cutoff value of 500 ng/mL (48, 49, 53). In our study the majority of IDDVT incidences (over 40% combined) were associated with the D-dimer levels in the ranges of 438–717 and over 717 ng/mL. Both these D-dimer ranges were significant predictors of IDDTV when adjusted for age and surgery method, with OR (95% CI) 2.32 (1.05–5.15) and OR (CI 95%) 2.93 (1.33–6.46), respectively. Levels below 438 ng/mL were not predictive of IDDVT. These results indicate that while D-dimer levels after surgery can quite reasonably predict the risk of IDDVT, the cutoff of elevated risk is not high, and may be just below 500 ng/mL. Further studies with larger cohorts of IDDVT patients are merited to define the exact cutoff of D-dimer levels.

Magnitude of surgical intervention have been shown to correlate with VTE recurrence (54).

Patients who underwent a high-risk procedure had a 34% incidence of DVT, while low-risk procedures were associated with only 11% incidence. Interestingly, our study indicates that Video-Assisted Thoracoscopic Surgery (VATS) (compared to thoracotomy) can increase the risk of IDDVT [OR (95% CI) 8.41 (1.25–56.75)]. Several large studies that addressed incidences of VTE in patients following VATS, reported a relative decrease in risk for vein thrombosis associated with this method, as compared to thoracotomy (55, 56). However, according to these studies, surgical approach cannot serve as an independent predictor of VTE (56). In our study, a small sample size results in a very large confidence interval, and thus greater uncertainty associated with the estimated OR. Further studies with higher sample sizes are needed to fully evaluate this potential effect of the type of surgery on the risk of IDDVT.

Postoperative bleeding is an important complication of pharmacoprophylaxis and may lead to serious complications and even death. Few studies have evaluated the risk of postoperative bleeding with or without pharmacoprophylaxis in an attempt to develop guidelines for proper prophylactic strategies for VTE in general surgery (non-cancer-related). These guidlines take into account patient and surgery-specific risks with consideration of the estimated bleeding risk. For instance, low-risk general abdominopelvic, gynecological and urological surgeries require either no prophylaxis or mechanical prophylaxis only. In contrast, minimal prophylaxis requirement for low-risk orthopedic, thoracic, neurologic, and vascular surgeries includes mechanical prophylaxis (such as compression stockings, active compression devices, and venous foot pumps) with/without pharmacological anticoagulation therapy (57). Recent randomized trials failed to reach a consensus on the best approach for treating IDDVT due to variations in study design, duration of treatment, and follow up (14, 23, 26–28, 58). The latest American College of Chest Physicians guidelines suggest anticoagulation therapy for IDDVT patients who are symptomatic or high-risk for extension (based on criteria such as serum D-dimer levels, extensive disease, or proximity to proximal veins, unprovoked DVT, malignancy, history of VTE, and inpatient status). All other cases are to be monitored by serial ultrasound examinations (21, 59). The efficacy of this approach in treating IDDVT is confirmed by a number of studies and trials (13, 26–28, 60–62). Longer treatment duration appears to be necessary in higher risk subgroups of IDDVT patients, but whether a treatment duration of 3 months is sufficient to prevent recurrences in these subgroups is unclear (63, 64). In our study, there was no difference in any of the adverse effects associated with DVT progression, such as bleeding, PE or mortality in patients, receiving half-dose (QD) of full dose (Q12H) of anticoagulation therapy. Both groups reported similar volumes of average daily drainage, and requirement for chest tube indwelling. Unsurprisingly, the duration of anticoagulation time differed significantly between Q12H and QD groups, with median 99 and 5 days, respectively. However, the treatment regimen correlated with the rate of medication required after the discharge. About one third of postoperative VTE events occur after discharge, and patients who are at increased risk may benefit from extended anticoagulation prophylaxis (35). Current guidelines recommend 7–10 days of total postoperative prophylaxis for cancer surgery patients. The majority (76.5%) of the patients in the Q12H group were treated with anticoagulation treatment at home, as compared to only 12.5% of patients in the QD group.

Our study has several limitations. The most significant factor, potentially impacting the outcomes of the study, is a small cohort of DVT and IDDVT patients. This paper is a retrospective study, which was originally an objective observation, not a clinical study of intervention measures. Additionally, the anticoagulation regimen of study participants was inconsistent and not standardized, especially the duration of anticoagulation.

In conclusion, in the recent study, we show that the D-dimer levels after surgery and the age of the patient are strong predictors of IDDVT following thoracic surgery. Half-dose therapeutic anticoagulation is not associated with any additional risks of adverse effects, and is as efficient as a full-dose regimen in preventing IDDVT progression. Half-dose therapy and ultrasound surveillance instead of prolonged full dose therapeutic anticoagulation might be an option for IDDVT patients after thoracic surgery. Further large randomized controlled studies with higher sample sizes are needed to evaluate the precise cut-off levels of post-operative D-dimer, and to develop a standard anticoagulation program for IDDVT patients with close follow-up.

## Data Availability Statement

The raw data supporting the conclusions of this article will be made available by the authors, without undue reservation.

## Ethics Statement

All procedures performed in studies involving human participants were in accordance with the ethical standards of the ethics committee of Shanghai Pulmonary Hospital and with the 1964 Helsinki declaration and its later amendments or comparable ethical standards (K20-454). Informed consent requirement was waived as this study was a retrospective study of consecutive patients that underwent thoracic surgery for diagnosed thoracic tumors of different etiology from July to November 2018.

## Author Contributions

YW and YL: conception and design. JY, GJ, YZhu, HW, and YZho: provision of study materials or patients. YL, JD, and LS: collection and assembly of data. YL: data analysis and interpretation. All authors: manuscript writing and final approval of manuscript.

## Conflict of Interest

The authors declare that the research was conducted in the absence of any commercial or financial relationships that could be construed as a potential conflict of interest.
